# On-field rehabilitation after anterior cruciate ligament injury in contact team sports: a scoping review of exercises, progression and key principles

**DOI:** 10.1136/bmjsem-2026-003344

**Published:** 2026-08-31

**Authors:** Anne Fältström, Timmy Gustafsson, Anna Hermansen, Sofi Sonesson

**Affiliations:** 1 Unit of Physiotherapy, Department of Health, Medicine and Caring Sciences, Linköping University, Linköping, Sweden; 2 Region Jönköping County, Rehabilitation Centre, Ryhov County Hospital, Jönköping, Sweden

**Keywords:** ACL, Rehabilitation, Sports rehabilitation programs, Contact sports, Sports & exercise medicine

## Abstract

**Objectives:**

To map and summarise the existing scientific literature on on-field rehabilitation (OFR) exercises, progression and principles supporting athletes’ return to contact team sports after anterior cruciate ligament (ACL) injury.

**Design:**

Scoping review.

**Data sources:**

PubMed, CINAHL, AMED, Cochrane, SPORTDiscus, PEDro, Scopus and Embase Library were searched in April 2025.

**Eligibility criteria for selecting studies:**

Sources of any design published between 2010 and 2025 that described or evaluated OFR for athletes returning to contact team sports after ACL injury or reconstruction, following Preferred Reporting Items for Systematic Reviews and Meta-Analyses Extension for Scoping Reviews (PRISMA-ScR) guidelines; studies involving athletes of any age, sex or competition level were eligible.

**Results:**

The search identified 6880 records; 38 sources met the inclusion criteria. Most sources were descriptive and focused predominantly on football, with limited representation of basketball, rugby and ice hockey. OFR was commonly described using multistage (2–5 steps) frameworks progressing from linear running and controlled drills to multidirectional movements, sport-specific skills and practice simulations. Progression was primarily criterion-based rather than time-based, guided by minimal pain or swelling, restored knee range of motion, adequate strength and limb symmetry, and tolerance to running loads. Several articles applied the control–chaos continuum. Collaboration between physiotherapists, coaches and athletes was emphasised, although roles, responsibilities and organisational structures were rarely described.

**Conclusion:**

The current OFR literature after ACL injury is largely descriptive, emphasising staged, criterion-based progression and multidisciplinary collaboration. However, actionable guidance on roles, responsibilities and organisational structures is minimal, underscoring the need for empirical studies to establish and evaluate standardised OFR practices.

**Trial registration number:**

10.17605/OSF.IO/T6UN2.

**WHAT IS ALREADY KNOWN**

- Late-stage rehabilitation after an anterior cruciate ligament injury is essential for restoring sport-specific movement patterns and performance, including sprinting, agility, intermittent endurance, shooting and jumping.
- Clinical rehabilitation protocols up to the point of return-to-field are well described and evaluated, but limited guidance exists on how to transition from controlled clinical settings to on-field rehabilitation (OFR) and how to structure progression strategies for team-sport athletes.
- Without well-structured OFR, there is a critical gap between physiotherapist-led rehabilitation and the complex, dynamic demands of team-sport environments.

**WHAT ARE THE NEW FINDINGS**

- OFR exercises are predominantly reported for football players, with limited evidence from basketball, rugby and ice hockey. These exercises are commonly described using multistage (2–5 steps) frameworks progressing from linear running and controlled drills to multidirectional movements, sport-specific skills and practice simulations.
- Exercise progression is predominantly criterion-based, advancing athletes from high-control to high-chaos environments, often structured using the control–chaos continuum. Progression is typically guided by minimal pain or swelling, restored knee range of motion, adequate strength and limb symmetry, and tolerance to increased running loads, rather than time elapsed.
- OFR principles emphasise interdisciplinary collaboration between physiotherapists, coaches and athletes. However, specific roles, responsibilities and organisational structures are rarely described.

## Introduction

 Evidence-based rehabilitation protocols after anterior cruciate ligament (ACL) injury are essential to optimise knee function, prevent secondary injuries and facilitate a safe return to sport (RTS).^1^ Rehabilitation principles after ACL injury or ACL reconstruction (ACLR) are similar and are divided into early stage (with range of motion and weightbearing exercises), mid-stage (with improved load tolerance and strength) and late-stage rehabilitation with sport-specific training.^2–4^ For athletes aiming to RTS, sport-specific on-field rehabilitation (OFR) training is a crucial component of late-stage recovery.^5^ OFR marks the transition from controlled gym-based rehabilitation to the dynamic competitive team environment.^6^ It plays a pivotal role in restoring sport-specific movement patterns and performance capabilities, including sprinting, agility, intermittent endurance, shooting and jumping.^7^ OFR is generally introduced during the mid-phase to late-phase of rehabilitation^5 8 9^ and is designed to bridge the gap between physiotherapist-led clinical rehabilitation and the demands of competitive play.^5 8 9^

Despite its importance, OFR remains poorly defined and underevaluated compared with earlier phases of ACL rehabilitation. Limited guidance exists on structured progression, exercise selection and criteria for advancing athletes towards RTS. This lack of clarity leads to inconsistent practices and creates a gap between clinical rehabilitation and the complex demands of competitive play. This scoping review aims to map and summarise the existing scientific literature on OFR exercises, progression and principles designed to support athletes returning to contact team sports after ACL injury. The findings will provide practical insights to support evidence-informed rehabilitation and facilitate a safe and successful RTS.

## Methods

### Protocol and registration

This scoping review adhered to the Preferred Reporting Items for Systematic Reviews and Meta-Analyses Extension for Scoping Reviews (PRISMA-ScR) guidelines,^10^ with a protocol registered on the Open Science Framework on 7 April 2025, before data extraction (https://osf.io/jv3d8, DOI:10.17605/OSF.IO/T6UN2). The methodological framework in five stages proposed by Arksey and O’Malley^11^ was used. These stages include (1) identification of the research question; (2) identification of relevant studies; (3) study selection; (4) data charting and (5) collating, summarising and reporting the results. The optional stakeholder-consultation stage was not undertaken, consistent with the exploratory aim of the scoping review.

### Review questions

Our research questions were

1. What OFR programmes and exercises aimed at supporting athletes returning to contact team sports after ACL injury are described in the literature?
2. What strategies and criteria are used to ensure progression during the rehabilitation process?
3. What are the rehabilitation principles and important elements described in the literature?

### Eligibility criteria

The inclusion criteria were sources that reported on OFR for athletes returning to contact team sports after ACL injury or on OFR for the lower extremity when applicable to knee joint injuries. Eligible sources were research sources published in English or Swedish between 2010 and 2025 available in full text. We included studies involving athletes of any age, sex and sport level (professional or competitive recreational), who were recovering from knee injuries, ACL injury or had undergone ACLR with any graft type. All study designs were considered, including meta-analyses, systematic reviews, scoping reviews, literature reviews, randomised controlled trials (RCTs), cohort studies, case–control studies, surveys, guidelines, editorials, commentaries, frameworks or technical reports, book chapters and expert opinions. Exclusion criteria were sources focusing on primary prevention of ACL injuries or rehabilitation programmes for specific injuries unrelated to the ACL.

### Information sources

A literature search was performed in eight electronic databases: PubMed, CINAHL, AMED, Cochrane, SPORTDiscus, PEDro, Scopus and Embase Library. To ensure completeness, we also screened the reference lists of all included sources to identify additional relevant publications.

### Search procedure

A systematic search strategy was developed, combining relevant keywords and Medical Subject Headings (MeSH). The search terms, combinations of search terms and the full electronic search strategy for the eight databases are presented in the online supplemental material 1. Search filters included publication years 2010–2025, and English or Swedish language. The search strategy was tailored to each database, adjusting to account for variations in indexing structures. On 14 March 2025, we conducted a librarian-assisted test search in one database (PubMed), before the final search on 23 April 2025 (online supplemental material 1).

### Selection process

All records were downloaded to EndNote (V.2025.1), and duplicates were removed. The remaining records were then exported to the Rayyan reference management platform (https://new.rayyan.ai/). Two independent reviewers (AF and TG) screened all titles and abstracts for eligibility based on the inclusion criteria. Before the selection process began, a pilot session took place to ensure alignment among the participating authors. In cases of disagreement between the two reviewers regarding eligibility, discussions were held until a consensus was reached. Full-text sources were retrieved for sources that were potentially relevant or where the eligibility was uncertain and were independently reviewed by the two reviewers. In cases of disagreement regarding eligibility, discussions were held until consensus was reached or by involving an additional reviewer (SS).

### Data extraction

Data extraction was guided by the research question and performed using a standardised extraction form developed specifically for this review and implemented in Excel. Both reviewers (AF and TG) independently extracted data from an initial sample of 10 sources to ensure consistency in how data were interpreted and recorded. The extracted data were compared, and any discrepancies were discussed until consensus was reached, which helped refine and clarify the extraction procedure. The remaining data were extracted by one author (AF). The following information was extracted: authors, year of publication, country of origin, sources of evidence (study design), level of evidence (I–V),^12^ a description of the OFR, target condition, sport level, progression, principles related to OFR and the RTS process, participant and intervention details (depending on study design) (online supplemental material 2). Level of evidence is reported in the table text (online supplemental table S1 and S2) or included in columns (online supplemental table S3). Participant characteristics and intervention details are reported where applicable (online supplemental table S3).

### Data synthesis

Data synthesis focused on systematically categorising, mapping and summarising themes and concepts across the included sources.^11^ An inductive approach was used, whereby themes were identified through iterative reading and comparison of the extracted data. Similar concepts were grouped into descriptive thematic categories, including staged frameworks, how OFR was structured, progression of exercises, criteria for progression and principles related to organisational aspects and healthcare roles. Results were synthesised to map the extent and characteristics of the sources and were presented using frequency counts, tables, figures and narrative summaries. The goal of this review was to provide an overview of the available literature and highlight any trends, gaps or commonalities in sport-specific OFR programmes rather than to conduct a detailed meta-analysis. Due to the exploratory nature of this review, assessments of bias and study quality were not included.^11^

## Results

### Sources of evidence

**Figure 1:**
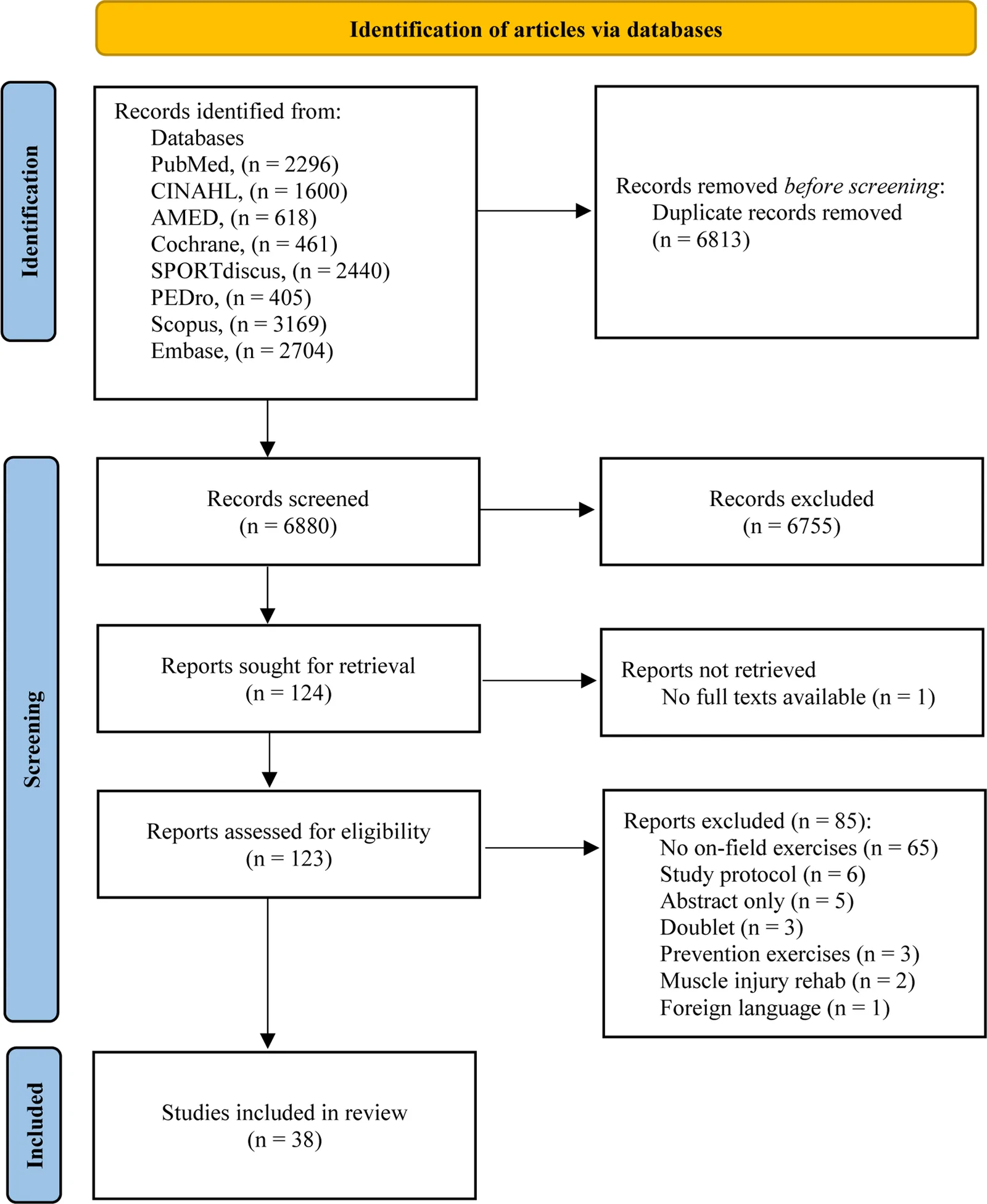
Preferred Reporting Items for Systematic Reviews and Meta-Analyses Extension for Scoping Reviews (PRISMA-ScR) flowchart showing the database search results, the number of records screened, reasons for exclusions and the final number of sources included in the scoping review.

The database searches yielded 6880 records. After screening titles and abstracts, the full text of 124 sources was assessed; 38 sources met the inclusion criteria and were summarised in text and tables. Snowballing of reference lists did not yield any additional sources. The study identification, screening and selection process is illustrated in a PRISMA-ScR flowchart (figure 1).

### Characteristics of sources of evidence

Publication years ranged from 2012 to 2025. 18 of the sources (48%) were from Europe, 13 (34%) from the USA, 5 (13%) from Australia and 2 (5%) from Asia. The types of sources were as follows: 12 clinical commentaries (32%),^9 13–23^ 6 book chapters (16%),^24–29^ 5 case reports (13%),^30–34^ 4 case series (11%),^35–38^ 3 cohort studies (8%),^39–41^ 3 frameworks or technical reports (8%),^42–44^ 2 (5%) RCTs,^45 46^ 1 literature review (3%),^47^ 1 editorial (3%)^48^ and 1 survey study (3%)^49^ (table 1). The level of evidence was IV–V in 27 (84%) of the 32 articles. No study was categorised as level I, and six sources were book chapters. The sources varied in depth, ranging from minimal reports on OFR exercises^19 40^ to highly comprehensive reports.^16 17 28^

**Table 1:** Characteristics of the sources reporting on on-field rehabilitation after anterior cruciate ligament injury

Variable	Category	n (%)
Country of origin	Europe	18 (48%)
	The USA	13 (34%)
	Australia	5 (13%)
	Asia	2 (5%)
Type of source	Clinical commentaries	12 (32%)
	Book chapters	6 (16%)
	Case reports	5 (13%)
	Case series	4 (11%)
	Cohort study	3 (8%)
	Framework/technical reports	3 (8%)
	Randomised control trials	2 (5%)
	Literature review	1 (3%)
	Editorial	1 (3%)
	Survey study	1 (3%)
Sports represented*	Football	18 (46%)
	Cutting/pivoting sports	7 (18%)
	Basketball	5 (13%)
	Rugby	2 (5%)
	American football	1 (3%)
	Australian football	1 (3%)
	Ice hockey	1 (3%)
	Not reported	4 (10%)
Level of play	Elite	11 (29%)
	Competitive level or any level	4 (11%)
	High school or amateur levels	2 (5%)
	Not reported	21 (55%)
Description of on-field rehabilitation	Figures/pictures	17 (45%)
	Video	14 (37%)
	Descriptive text/tables	7 (18%)

* One source included both football and basketball.

An overall summary of the clinical commentaries,^9 13–23^ book chapters,^24–29^ frameworks or technical reports,^42–44^ the survey study,^49^ the literature review^47^ and the editorial^48^ are presented in online supplemental table S1. An overall summary from the five case reports involving three male and two female athletes^30–34^ is presented in online supplemental table S2. The nine remaining prospective cohort studies,^39–41^ case series^35–38^ and RCTs^45 46^ are presented in online supplemental table S3. Of these nine prospective cohort studies, six included both male and female participants^36–39 41 45^ and three studies included only men.^35 40 46^

The sources reported and demonstrated examples of OFR exercises with figures/pictures (17 sources, 45%).^9 13–15 18–22 24–28 37 45 49^ 14 (37%) sources included videos.^9 14 16 17 23 31 32 34 39 42 43 46–48^ Seven sources (18%) included only descriptive text/tables of the exercises.^33 35 36 38 40 41 44^ Most of the OFR exercises were reported for football players (18 sources, 46%).^9 13 14 19 20 22 27 28 33–36 40 43 44 46 48 49^ Five sources (13%) reported OFR for basketball players^17 21 24 25 28^ and two (5%) for rugby players.^15 32^ In addition, one source focused on ice hockey,^23^ one on American football^16^ and one on Australian football.^31^ Some sources did not specify the sport beyond general contact sports involving cutting and pivoting^29 30 37 39 41 42 45^ or did not report any specific sport.^18 26 38 47^ The level of play was not reported in 21 sources (55%).^9 13–16 18 19 23–30 37 38 41 42 45 47^ Among the sources that specified the level of play, 11 sources (29%) focused on elite-level participants,^20–22 31–34 44 46 48 49^ 4 sources (11%) were conducted at a competitive level or any level^17 35 36 43^ and 2 sources (5%) examined high school or amateur levels^39 40^ (table 1). Two sources focused on the progression of a kicking programme to support clinicians in allowing football players to safely return to the demands of their sport.^13 44^ Two sources focused specifically on on-field running and sprinting exercises.^19 40^

### Summary of the findings

Overall, the findings of this scoping review are primarily based on sources representing low levels of evidence (levels IV–V) and book chapters. A small number of higher-level studies (five level II studies, including prospective cohort studies and RCTs) reported improvements in hop test limb symmetry,^37 39 45^ muscle strength^36 37 39^ and patient-reported outcome measures^36 37 41 45^ following OFR programmes, as well as facilitation of RTS^35 38 46^ and low reinjury rates.^38 41 46^

The results are presented in three main sections: exercises, criteria used to ensure progression and principles for OFR. In this scoping review, the number of sources supporting each finding can be identified through the cited references accompanying each result statement.

#### On-field rehabilitation exercises

**Figure 2:**
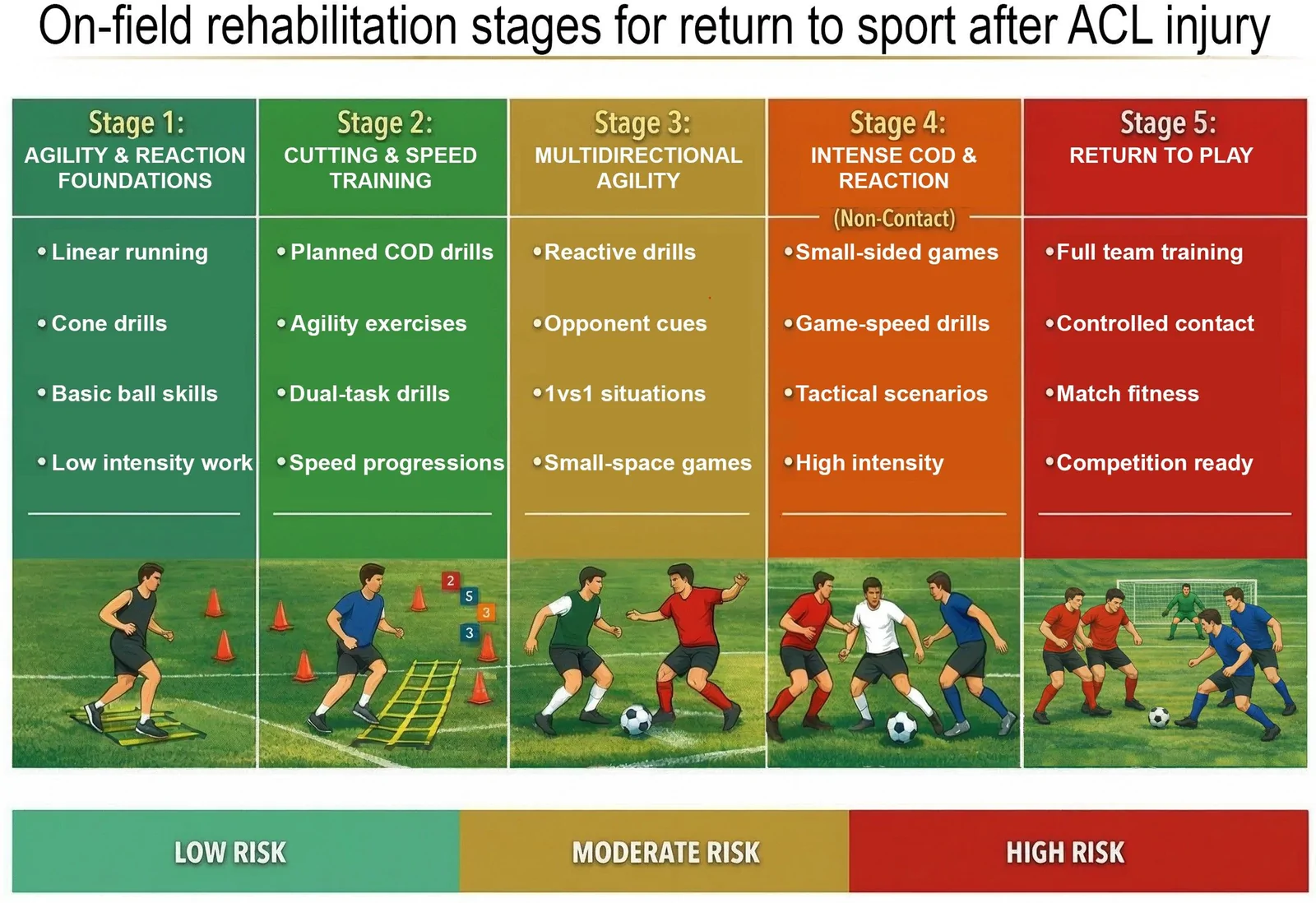
Synthesis of on field rehabilitation stages for return to sport after ACL injury or ACL reconstruction, generated with Copilot and Gemini based on the included sources. The risk of (re)injury is low in the initial stage and increases progressively as intensity and knee specific demands rise. ACL, anterior cruciate ligament; COD, change of direction.

The OFR exercises focused on developing sport-specific skills that exposed athletes to repeated knee impact moments and addressed the demands of activities such as agility, change of direction, cutting, pivoting and coordination. These exercises emphasised the reintroduction of essential sport-specific skills, which included passing, hand–eye coordination drills, hop and landing practice, and reaction activities, often involving balls. All the OFR exercises were performed in a progressive manner. The literature commonly described OFR within a staged framework, ranging from two,^42^ three,^16 17 27^ four^14 23 30^ to five phases, typically structured as (1) linear movement, (2) multidirectional movement, (3) sport-specific technical skills, (4) sport-specific movement and (5) practice simulation.^9 21 25 26 31 33 35 36 38 46 48^ The progression from restricted to unrestricted training, reintroducing sport-specific skills, often followed the control–chaos continuum^20–22 31–34 43 48 49^ developed by Taberner *et al*^48^ in 2019. The control–chaos continuum, mostly described in expert-based and conceptual sources, was presented as a flexible framework that gradually increased running loads while adding perceptual and neurocognitive challenges through specific drills to ensure specificity and athlete engagement. Training was designed to mimic team demands in their environments and align with sport-specific contexts, taking into account the training phase and individual needs. Ultimately, the OFR increasingly resembled typical team training, incorporating worst-case scenario training (full contact plus position-specific movement pattern drills, additional contact drills, additional dynamic skill drills and running conditioning), followed by limited playing time in matches and finally progressing to full match play (figure 2).

#### Criteria used to ensure OFR progression

The OFR programme was described as first establishing personal goals for each athlete, and that the process would be conducted collaboratively by the athlete and the treating physiotherapist.^27^ Implementing and progressing through the OFR phases was guided by function achieved rather than time elapsed. Important aspects to consider were when the athlete felt comfortable, had no perceived knee instability, no or minimal symptoms such as pain (eg, visual analogue scale <3 of 10), no fear of reinjury, felt coordinated and tolerated the exercises without swelling or reduced range of motion. Good function included good knee control, movement quality, balance and strength. Acceptable strength asymmetry thresholds between limbs varied in the sources and by OFR phase, with higher asymmetry (≤20–30%) permitted in earlier phases and stricter criteria, including full symmetry (0%), applied in later phases or at RTS online supplemental tables S1–S3. This was often evaluated using different functional tests, including, for example, strength, balance, hop, running and on-field tests.^9 13–17 19 21–23 25–31 33 34 36–41 43–48^

The exercises were largely selected and progressed based on practitioners’ own experience.^49^ Wearable technology (ie, GPS) was commonly used at the elite level to support OFR decision-making.^9 19 31–35 43 46 48 49^

#### Principles for on-field rehabilitation

The reviewed sources did not provide in-depth descriptions of the principles related to economic considerations or the role of different healthcare providers in OFR. However, collaboration among the medical team, athlete and coach was reported as essential in expert-based sources.^14 20 22 24 42^ Proper pacing and open communication during the early phases of rehabilitation were described as important for synchronising everyone’s expectations.^33^ Initially, athletes were advised to perform agility progressions under the supervision of their physiotherapist or trainer to allow monitoring of movement patterns.^26^ However, athletes often followed advice from coaches, who were described as having more experience in training healthy athletes than those recovering from ACL injury.^36^ Coaches become particularly important in the final stages, whereas doctors mainly contributed through the diagnosis and medical clearance.^49^

## Discussion

This scoping review identified 38 sources with OFR exercises designed to support athletes returning to contact team sports after an ACL injury. OFR was predominantly described in football and typically followed a multistage progression from highly controlled to increasingly chaotic and sport-specific activities, guided by criterion-based rather than time-based progression. Collaboration between physiotherapists, coaches and athletes is emphasised, although roles, responsibilities and organisational structures are often poorly defined.

In the reviewed material, OFR and RTS were portrayed as individualised and structured processes that bridge the gap between general rehabilitation and full participation in team sport. OFR typically progressed from controlled to dynamic and chaotic activities, with gradual increases in running load and incorporation of perceptual and neurocognitive demands.^9 48 50^ Progression from restricted to unrestricted training was commonly guided by multi-stage frameworks, including the control–chaos continuum, emphasising restoration of movement quality, progressive physical loading and sport-specific skill development.^9 27 48 50^ This progression from simple unidirectional movements to complex, multidirectional, sport-specific tasks was described as important for rebuilding athlete confidence and gradually increasing external knee loading.^27^ However, these frameworks were largely based on expert opinion and case studies, underscoring the need for more robust empirical validation. Articles adding OFR to the traditional protocols with prospective designs showed promising results regarding the RTS rate^35 38^ or time to RTS,^46^ reinjury rate,^38 41 46^ strength,^36 37 39^ hop capacity,^37 39 45^ aerobic threshold^35 36 40^ and patient-reported outcome measures.^36 37 41 45^ Although the number of such studies remains limited, their findings suggest that structured OFR may have a meaningful influence on both performance and safety during the RTS transition.

Football, one of the biggest sports in the world in terms of the number of practitioners,^51^ was a primary focus. Nearly half of the sources reviewed focused on OFR for football players. Basketball, another worldwide sport, was highlighted in 13% of the sources. Many sources discussed OFR in general without specifying a particular sport, although they often referred to contact sports that involved cutting and pivoting movements. Team sports differ in physical demands based on the field size, position, level of play and sex. Most share fundamental motor skills; therefore, OFR should begin with low-demand skill relearning and progress to movements imposing greater challenges on knee stability.^42^ In the later stages of rehabilitation, the programme was customised for each participant’s sport. For instance, volleyball players concentrated on jumping exercises, whereas football players focused on cutting and agility tasks.^38^ Notably, we found only one article that specifically addressed OFR for ice hockey players after ACLR.^23^ The OFR differed significantly, particularly concerning the playing surface (eg, grass vs ice).

OFR exercises depending on the level of play were not reported in two-thirds of the sources, and one-fourth focused on elite-level athletes. The significance of the level of play in the context of OFR may be minor, even if the circumstances differ a lot. The elite player has access to a medical team that can attend to the OFR in a manner far beyond that available to the amateur player. The principles governing OFR exercises should remain consistent regardless of the athlete’s level. The focus should be on individualising the exercises based on the athlete’s condition and knee status.^3 24 26^ A recently published narrative review^50^ outlined postoperative ACLR progression in elite and professional athletes, providing a general framework applicable across sports, although basketball, American football, football and skiing were emphasised.

None of the sources featuring descriptive or clinical commentaries specifically addressed whether there were, or should be, sex differences in OFR. Other sources included both sexes and did not differentiate OFR exercises based on sex. This suggests that OFR may be more dependent on individual characteristics than on sex. Nevertheless, it raises an interesting question, given the different physical conditions that exist in contact sports based on sex.

The OFR was described using text, images and even videos in one-third of the material, which could be beneficial for demonstrating the exercises. Simply describing the exercises, such as zigzag running with and without a ball, lateral side steps with and without a ball, and 45° direction changes, might be difficult for both athletes and coaches to fully grasp. Including pictures or videos alongside the text will greatly enhance understanding of the exercises.

In our review, we found that the roles, responsibilities and organisational structures of healthcare providers in the process of OFR were not well described. Clearer role definitions and organisational structures are needed to ensure coordinated OFR, particularly during transitions from physiotherapist-led to coach-led phases. This can be attributed to the fact that OFR is often managed by the athlete themselves, with guidance from their regular physiotherapist remotely and possibly some input from their coach. Collaboration between physiotherapists and athletic trainers can increase treatment sessions, and clear progression criteria could enhance care consistency, regardless of provider.^38^ The communication between the athlete, physiotherapist and coach should be an important factor in this phase of rehabilitation.^52–54^ Coaches and physiotherapists need enhanced knowledge to assist athletes in RTS after ACL injury.^53^ The importance of different monitoring tools to inform decision-making among English practitioners responsible for the design and implementation of OFR ranked verbal communication highest (100% positive), followed by functional/clinical experience/expertise (94% positive), self-reported measures (86% positive), wearable technology (91% positive) and video (23% positive).^49^ Structuring the OFR exercises and progression could facilitate communication between healthcare providers.

### Clinical implications

This scoping review identifies several practical considerations that may improve clinical decision-making during OFR after ACL injury in contact team sports. The literature suggests that OFR is most effective when progression is structured within a stepwise, criterion-based framework rather than a time-driven schedule. Clinicians may therefore consider prioritising knee function—pain and swelling status, range of motion, strength, limb symmetry and tolerance to running loads—when determining readiness to advance toward multi-directional drills, sport-specific skills and practice-based scenarios.

For practitioners, one key implication is the need to design OFR that deliberately increases movement complexity and contextual demands to better prepare athletes for the unpredictable nature of contact team sports. Another important consideration is the role of interdisciplinary collaboration: although cooperation between physiotherapists, coaches and athletes is consistently emphasised, the literature offers limited guidance on how responsibilities should be organised. Teams may therefore benefit from actively defining roles, communication routines and shared criteria for progression.

By summarising available OFR exercises, advancement criteria and proposed frameworks, this review provides clinicians and performance staff with a foundation for developing more coherent, transparent and function-oriented OFR practices. These insights may support safer and more consistent decision-making during the return-to-sport process for athletes recovering from ACL injury.

### Strengths and limitations

To the best of our knowledge, this scoping review is the first to focus on OFR exercises designed to support athletes returning to contact team sports after ACL injury. Scoping reviews are useful for mapping broad and complex research areas, identifying available evidence, clarifying key concepts and highlighting knowledge gaps.^11 55^ One strength of this review is the comprehensive librarian-assisted search conducted across eight databases and predefined eligibility criteria, which enabled a thorough overview of the existing literature. Furthermore, our earlier qualitative work with athletes after ACLR,^52^ who expressed a clear need for OFR guidance, supports the relevance of the topic and aligns with step 6 of Arksey and O’Malley’s framework.^11^

Several limitations must be acknowledged. We did not report inter reviewer reliability statistics. While reporting such statistics may enhance methodological transparency, this is not routinely performed in scoping reviews and is not required by PRISMA-ScR guidelines. A critical appraisal of individual sources of evidence was not performed, as the objective of this scoping review was to map existing literature rather than to assess study quality. While this approach is consistent with scoping review methodology,^11^ it means that the findings may be informed by studies of varying quality. Among the 38 sources in our review, 6 were book chapters and only 2 were RCTs. Much of the available guidance consists of expert opinion, case studies or conceptual frameworks. Evidence for late-stage OFR, where the focus shifts to reducing secondary injury risk, enhancing tissue tolerance and improving psychological readiness, is particularly limited, underscoring the need for more prospective cohort studies and RCTs.^30^ We did not evaluate RTS criteria or batteries of functional tests because these have been extensively reviewed elsewhere.^56–62^

The study protocol specified dual independent data extraction. However, due to resource constraints, extraction was primarily conducted by the first author, with a random sample of 10 sources independently checked by a second reviewer to assess consistency. This deviation from the protocol was not registered as a formal amendment in the Open Science Framework and is acknowledged as a limitation. The potential impact is mainly an increased risk of random extraction errors rather than systematic bias, which is mitigated by reviewer calibration and the descriptive aim of this scoping review. Because we focused on ACL injuries and OFR in contact sports, generalisability to individual sports may be limited, although the findings may be relevant to other lower extremity injuries. Restricting inclusion to English and Swedish sources may have introduced publication bias. In addition, only published sources were included; conference abstracts, organisational documents and informal resources (eg, webpages, training manuals) were excluded, meaning that innovative but undocumented OFR practices may not have been captured.

## Conclusions

The current literature describes OFR using consistent themes of staged, criterion-based progression, with most examples focused on football and a lack of exercises tailored to ice hockey. Guidance regarding roles, responsibilities and organisational structures within OFR remains limited. The existing evidence is mainly descriptive and predominantly comprises expert opinion, clinical commentaries, case reports and technical reports. Future research should prioritise prospective study designs to evaluate how different OFR exercises influence key outcomes such as reinjury rates, RTS rate and patient-reported outcome measures, including quality of life.

## Supplementary material

10.1136/bmjsem-2026-003344 online supplemental file 1

10.1136/bmjsem-2026-003344 online supplemental file 2

## Data Availability

All data relevant to the study are included in the article or uploaded as supplementary information.
