# Supplementary material for: On-field rehabilitation after anterior cruciate ligament injury in contact team sports: a scoping review of exercises, progression and key principles
Source: BMJ Open Sport Exerc Med. 2026 Aug 31;12(3):e003344. doi: 10.1136/bmjsem-2026-003344 (PMC13536009; doi:10.1136/bmjsem-2026-003344)
Supplement: Supplementary file 1 [file bmjsem-12-3-s001.pdf]

Supplemental material 1

Table 1. Search terms, how the search was conducted, and how the different blocks were combined.

**Block 1:**<br>**Anterior cruciate ligament**<br>**AND →**	**Block 2a:**<br>**On-field**<br>**OR →**	**Block 2b:**<br>**Contact sports**<br>**OR →**	**Block 2c:**<br>**Return to sport**
Athletic injuries[mesh]<br>Anterior cruciate ligament[mesh]<br>Anterior cruciate ligament injuries[mesh]<br>Anterior cruciate ligament reconstruction[mesh]<br><br>Anterior cruciate ligament[tiab]<br>ACL[tiab]<br>ACLR[tiab]<br>Injury*[tiab]<br>Knee[tiab]<br>Lower extremity[tiab]<br>Lower limb[tiab]	Court[tiab]<br>End-phase[tiab]<br>Field[tiab]<br>Field-based[tiab]<br>Field rehabilitation[tiab]<br>Field-side[tiab]<br>Final phase[tiab]<br>Late-stage[tiab]<br>On-court[tiab]<br>On-field[tiab]<br>On-pitch[tiab]<br>Pitch[tiab]<br>Pitch-based[tiab]<br>Pitch-side[tiab]<br>Team training[tiab]<br>Sport-specific[tiab]<br><br>**Combined with:**<br>**Rehab*[tiab]**<br>**Rehabilitation[mesh]**	Basketball[tiab]<br>Basketball[mesh]<br>Contact sport*[tiab]<br>Ice hockey[tiab]<br>Lacrosse[tiab]<br>Field hockey[tiab]<br>Hockey[mesh]<br>Floorball[tiab]<br>Football[tiab]<br>Football[mesh]<br>Handball[tiab]<br>Hurling[tiab]<br>Rugby[tiab]<br>Rugby[mesh]<br>Soccer[tiab]<br>Soccer[mesh]<br><br>**Combined with:**<br>**Rehab*[tiab]**<br>**Rehabilitation[mesh]**	Return to sport[mesh]<br>Return to sport*[tiab]<br>Resumption of Sport*[tiab]<br>Return to play[tiab]<br>Return to performance[tiab]<br>Return to competition[tiab]<br>Return to training[tiab]<br>Return-to-sport Training[tiab]<br><br>**Combined with:**<br>**(Rehab*[tiab])**<br>**(Rehabilitation[mesh])**

ACL, anterior cruciate ligament; ACLR, anterior cruciate ligament reconstruction; Mesh, medical subject headings; Tiab, title/abstract.

*Truncation (*): Searches for word variants from the place in the word where *has been placed. Sport* therefore matches sport, sports, sporting.

The full electronic search strategy for the 8 databases at April 23 at 9 am.

**Search strategy – PubMed**		
Nr	Block	Counts
1	"Anterior Cruciate Ligament Reconstruction"[Mesh] OR "Anterior Cruciate Ligament"[Mesh] OR "Anterior Cruciate Ligament Injuries"[Mesh] OR "Athletic Injuries"[Mesh] OR “anterior cruciate ligament”[tiab] OR ACL[tiab] OR ACLR[tiab] OR ((knee[tiab] OR “lower extremity”[tiab] OR “lower limb”[tiab]) AND injur*[tiab])	99,389
2	Court[tiab] OR “End-phase”[tiab] OR Field[tiab] OR “final phase”[tiab] OR “Late-stage”[tiab] OR pitch[tiab] OR “team training”[tiab] OR “sport-specific”[tiab]	1,160,891
3	Basketball[tiab] OR Basketball[mesh] OR “Contact sport*”[tiab] OR hockey[tiab] OR lacrosse[tiab] OR hockey[mesh] OR Floorball[tiab] OR Football[tiab] OR Football[mesh] OR Handball[tiab] OR hurling[tiab] OR rugby[tiab] OR rugby[mesh] OR Soccer[tiab] OR soccer[mesh]	42,140
4	“return to sport”[mesh] OR “return to sport*”[tiab] OR “Resumption of Sport*”[tiab] OR “return to play”[tiab] OR “return to performance”[tiab] OR “return to competition”[tiab] OR “return to training”[tiab]	10,522
5	2 OR 3 OR 4	1,205,742
6	(Rehab*[tiab]) OR (Rehabilitation[mesh])	558,960
7	5 AND 6	22,128
8	1 AND 7	2,897
9	(2010/1/1:3000/12/12[pdat]) AND (English[Filter] OR Swedish[Filter])	FILTER
10	8 AND 9	2,296

Search strategy Cumulative Index to Nursing and Allied Health Literature (CINAHL)		
Nr	Block	Counts
1	(MH "Anterior Cruciate Ligament") OR (MH "Anterior Cruciate Ligament Injuries") OR (MH "Anterior Cruciate Ligament Reconstruction") OR (MH "Athletic Injuries+")	38,702
2	"anterior cruciate ligament" OR ACL OR ACLR	18,684
3	(knee OR "lower extremity" OR "lower limb") AND injur*	30,530
4	1 OR 2 OR 3	62,062
5	Court OR "End-phase" OR Field OR "final phase" OR "Late-stage" OR pitch OR "team training" OR "sport-specific"	173,995
6	"return to sport*" OR "resumption of sport*" OR "return to play" OR "return to performance" OR "return to competition" OR "return to training"	6,627
7	(MH "Basketball") OR (MH "Contact Sports") OR (MH "Hockey") OR (MH "Lacrosse") OR (MH "Football") OR (MH "Handball") OR (MH "Rugby") OR (MH "Soccer")	16,516
8	basketball OR "contact sport*" OR hockey OR lacrosse OR floorball OR football OR handball OR hurling OR rugby OR soccer	25,203
9	7 OR 8	25,203
10	5 OR 6 OR 9	201,109
11	(MH "Rehabilitation")	18,249
12	rehab*	217,065
13	11 OR 12	217,065
14	10 AND 13	10,414
15	4 AND 14	2,362
16	15 AND Filter: peer review, 2010/01-, English	1,600

Search strategy Allied and Complementary Medicine Database (AMED)		
Nr	Block	Counts
1	"anterior cruciate ligament" OR "athletic injur*" OR ACL OR ACLR	7,235
2	(knee OR "lower extremity" OR "lower limb") AND injur*	5,096
3	S1 OR S2	9,993
4	Court OR "End-phase" OR Field OR "final phase" OR "Late-stage" OR pitch OR "team training" OR "sport-specific"	8,098
5	"return to sport*" OR "resumption of sport*" OR "return to play" OR "return to performance" OR "return to competition" OR "return to training"	941
6	basketball OR "contact sport*" OR hockey OR lacrosse OR floorball OR football OR handball OR hurling OR rugby OR soccer	2,269
7	S4 OR S5 OR S6	10,795
8	rehab*	94,454
9	S7 AND S8	3,621
10	S3 AND S9	1,012
11	S9 AND Peer review, 2010/01-, English	618

Search strategy Cochrane		
Nr	Block	Counts
1	MeSH descriptor: [Athletic Injuries] explode all trees	1,011
2	MeSH descriptor: [Anterior Cruciate Ligament] explode all trees	836
3	MeSH descriptor: [Anterior Cruciate Ligament Injuries] explode all trees	1,284
4	MeSH descriptor: [Anterior Cruciate Ligament Reconstruction] explode all trees	751
5	("anterior cruciate ligament"):ti,ab,kw OR (ACL):ti,ab,kw OR (ACLR):ti,ab,kw	4,341
6	(knee):ti,ab,kw OR ("lower extremity"):ti,ab,kw OR ("lower limb"):ti,ab,kw	59,702
7	(injur*):ti,ab,kw	85,628
8	#6 AND #7	9,210
9	#1 OR #2 OR #3 OR #4 OR #5 OR #8	12,063
10	(court):ti,ab,kw OR ("end-phase"):ti,ab,kw OR (field):ti,ab,kw OR ("final phase"):ti,ab,kw OR ("late-stage"):ti,ab,kw OR (pitch):ti,ab,kw OR ("team training"):ti,ab,kw OR ("sport-specific"):ti,ab,kw	33,564
11	MeSH descriptor: [Basketball] explode all trees	339
12	MeSH descriptor: [Hockey] explode all trees	97
13	MeSH descriptor: [Football] explode all trees	401
14	MeSH descriptor: [Rugby] explode all trees	30
15	MeSH descriptor: [Soccer] explode all trees	1,155
16	(contact NEXT sport*):ti,ab,kw OR (basketball):ti,ab,kw OR (hockey):ti,ab,kw OR (lacrosse):ti,ab,kw OR (floorball):ti,ab,kw OR (football):ti,ab,kw OR (handball):ti,ab,kw OR (hurling):ti,ab,kw OR (rugby):ti,ab,kw OR (soccer):ti,ab,kw	5,204
17	#11 OR #12 OR #13 OR #14 OR #15 OR #16	5,204
18	MeSH descriptor: [Return to Sport] explode all trees	76
19	(return NEXT to NEXT sport*):ti,ab,kw OR (resumption NEXT of NEXT sport*):ti,ab,kw OR ("return to play"):ti,ab,kw OR ("return to performance"):ti,ab,kw OR ("return to competition"):ti,ab,kw OR ("return to training"):ti,ab,kw	697
20	#18 OR #19	697
21	MeSH descriptor: [Rehabilitation] explode all trees	55,590
22	(rehab*):ti,ab,kw	75,088
23	#21 OR #22	112,268
24	#10 OR #17 OR #20	38,779
25	#24 AND #23	3,589
26	#9 AND #25 with Cochrane Library publication date Jan 2010	473
27	Filter: English, Swedish	461

Search strategy SPORTDiscus		
Nr	Block	Counts
1	(DE "ANTERIOR cruciate ligament" OR DE "ANTERIOR cruciate ligament injuries" OR DE "ANTERIOR cruciate ligament surgery" OR DE "ANTERIOR cruciate ligament transplantation") OR (DE "SPORTS injuries" OR DE "ACHILLES tendinitis" OR DE "AEROBICS injuries" OR DE "AQUATIC sports injuries" OR DE "BASEBALL injuries" OR DE "BASKETBALL injuries" OR DE "BOXING injuries" OR DE "COMMOTIO cordis" OR DE "CRICKET injuries" OR DE "DELAYED onset muscle soreness" OR DE "EQUESTRIAN accidents" OR DE "FOOTBALL injuries" OR DE "GOLF injuries" OR DE "GYMNASTICS injuries" OR DE "HIKING injuries" OR DE "HOCKEY injuries" OR DE "HORSE sports injuries" OR DE "IN-line skating injuries" OR DE "JOGGING injuries" OR DE "JUDO injuries" OR DE "JUMPER'S knee" OR DE "KARATE injuries" OR DE "MARTIAL arts injuries" OR DE "MOTORSPORTS injuries" OR DE "NETBALL injuries" OR DE "RACKET game injuries" OR DE "RUGBY football injuries" OR DE "RUNNING injuries" OR DE "SKATEBOARDING injuries" OR DE "SOCCER injuries" OR DE "TENNIS injuries" OR DE "TURF toe" OR DE "VAULTING injuries" OR DE "VOLLEYBALL injuries" OR DE "WALKING (Sports) injuries" OR DE "WEIGHT training injuries" OR DE "WINTER sports injuries")	34,667
2	“anterior cruciate ligament” OR ACL OR ACLR	15,077
3	(knee OR “lower extremity” OR “lower limb”) AND injur*	29,904
4	1 OR 2 OR 3	56,448
5	Court OR “End-phase” OR Field OR “final phase” OR “Late-stage” OR pitch OR “team training” OR “sport-specific”	143,651
6	DE "SPORTS re-entry"	623
7	“return to sport*” OR “resumption of sport*” OR “return to play” OR “return to performance” OR “return to competition” OR “return to training”	5,548
8	6 OR 7	5,548
9	(DE "BASKETBALL" OR DE "CONTACT sports") OR (DE "HOCKEY") OR (DE "LACROSSE") OR (DE "FOOTBALL") OR (DE "HANDBALL") OR (DE "HURLING (Game)") OR (DE "RUGBY football") OR (DE "SOCCER")	132,535
10	basketball OR “contact sport*” OR hockey OR lacrosse OR floorball OR football OR handball OR hurling OR rugby OR soccer	363,664
11	9 OR 10	363,664
12	5 OR 8 OR 11	484,406
13	DE "REHABILITATION"	27,566
14	rehab*	119,068
15	13 OR 14	119,068
16	12 AND 15	10,560
17	4 AND 16	3,373
18	17 AND Filter: peer review, 2010/01-, English	2,440

Search strategy PEDro		
Nr	Block	Counts
1	"Anterior Cruciate Ligament" rehab*	153
2	ACL rehab*	129
3	ACLR rehab*	48
4	"knee injury" rehab*	54
5	"knee injuries" rehab*	8
6	"Athletic injury" rehab*	0
7	"athletic injuries" rehab*	2
8	"lower limb injury" rehab*	4
9	"lower limb injuries" rehab*	2
10	"lower extremity injury" rehab*	4
11	"lower extremity injuries" rehab*	1

Search strategy – Scopus		
Nr	Block	Counts
1	(TITLE-ABS-KEY("anterior cruciate ligament" OR "athletic injur*" OR "sport injur*" OR "acl" OR "aclr" OR ((knee OR "lower extremity" OR "lower limb") AND injur*)))	175,601
2	(TITLE-ABS-KEY(court OR "end-phase" OR field OR "final phase" OR "late-stage" OR pitch OR "team training" OR "sport-specific"))	7,358,777
3	(TITLE-ABS-KEY(basketball OR "contact sport*" OR hockey OR lacrosse OR floorball OR football OR handball OR hurling OR rugby OR soccer))	104,051
4	(TITLE-ABS-KEY("return to sport*" OR "resumption of sport*" OR "return to play" OR "return to performance" OR "return to competition" OR "return to training"))	13,985
5	2 OR 3 OR 4	7,457,789
6	(TITLE-ABS-KEY(rehab*))	512,236
7	5 AND 6	38,078
8	1 AND 7	4,123
9	(PUBYEAR AFT 2009) AND (LANGUAGE(English OR Swedish))	FILTER
10	8 AND 9	3,169

Search strategy – Embase		
Nr	Block	Counts
1	'anterior cruciate ligament reconstruction'/exp OR 'anterior cruciate ligament'/exp OR 'anterior cruciate ligament injury'/exp OR 'sport injury'/exp OR 'anterior cruciate ligament':ti,ab,kw OR 'acl':ti,ab,kw OR 'aclr':ti,ab,kw OR (('knee':ti,ab,kw OR 'lower extremity':ti,ab,kw OR 'lower limb':ti,ab,kw) AND 'injur*':ti,ab,kw)	127,694
2	'court':ti,ab,kw OR 'end-phase':ti,ab,kw OR 'field':ti,ab,kw OR 'final phase':ti,ab,kw OR 'late-stage':ti,ab,kw OR 'pitch':ti,ab,kw OR 'team training':ti,ab,kw OR 'sport-specific':ti,ab,kw	1,287,375
3	'basketball':ti,ab,kw OR 'basketball'/exp OR 'contact sport*':ti,ab,kw OR 'contact sport'/exp OR 'hockey':ti,ab,kw OR 'lacrosse':ti,ab,kw OR 'hockey'/exp OR 'lacrosse (sport)'/exp OR 'floorball':ti,ab,kw OR 'football':ti,ab,kw OR 'football'/exp OR 'handball':ti,ab,kw OR 'hurling':ti,ab,kw OR 'rugby':ti,ab,kw OR 'rugby'/exp OR 'soccer':ti,ab,kw OR 'soccer'/exp	49,227
4	'return to sport'/exp OR 'return to sport*':ti,ab,kw OR 'resumption of sport*':ti,ab,kw OR 'return to play':ti,ab,kw OR 'return to performance':ti,ab,kw OR 'return to competition':ti,ab,kw OR 'return to training':ti,ab,kw	14,010
5	2 OR 3 OR 4	1,341,155
6	'rehab*':ti,ab,kw OR 'rehabilitation'/exp	759,022
7	5 AND 6	30,000
8	1 AND 7	3,832
9	[2010 to 3000]/pd AND ('English':la OR 'Swedish':la)	FILTER
10	8 AND 9	2,704
