# Supplementary material for: On-field rehabilitation after anterior cruciate ligament injury in contact team sports: a scoping review of exercises, progression and key principles
Source: BMJ Open Sport Exerc Med. 2026 Aug 31;12(3):e003344. doi: 10.1136/bmjsem-2026-003344 (PMC13536009; doi:10.1136/bmjsem-2026-003344)
Supplement: Supplementary file 2 [file bmjsem-12-3-s002.pdf]

Supplemental material 2.

Table S1 The 24 clinical commentary, frameworks or technical reports, editorial, literature review and survey sources (level of evidence IV–V) reporting on-field rehabilitation exercises applicable for athletes with ACL injury

Author (year)	Country	Sources of evidence	Description of OFR	Sport	Target condition	Sport level	Progression	Criteria for progression	Principles in the OFR and RTS process
Allen et al. (2021)[43]	UK	Methodological framework	Text, tables, figures, pictures and videos	Football, applicable to sports such as rugby, basketball	Knee (ACL)	Elite, applicable for amateur/semi-professional	Control–chaos continuum model	Progression was based on medical/performance criteria: running load, technical skills, strength/power tests, pain/swelling <2/10, confidence (0–10), and perceived exertion feedback	Player-coach communication; coaches adjust constraints (pitch size, rules) to control load, variability, and match-relevant rehabilitation demands; GPS used
Armitage et al. (2024)[49]	UK	Survey study of 69 practitioners (75% response): 67% performance coaches, 33% medical staff, responsible for OFR design/implementation at English professional football clubs	Text, tables and figures	Football	Injuries in general	Elite	Progression was logical and consistent, supporting a continuum-based approach for controlled OFR tasks	OFR drills were largely selected and progressed based on own experience	OFR was multi-disciplinary; therapists led with input from strength, technical, and medical staff; GPS was commonly used
Arundale et al. (2020)[24]	USA	Book chapter	Text, tables, figures and pictures	Basketball	Injuries in general	NR	Basketball could start early in rehabilitation, progressing safely to sport-specific drills that build complexity and address injury challenges	Once basic movements were controlled, progressed by adjusting the environment, speed, intensity, perturbations, and cognitive load	Clear communication and collaboration involving therapists, coaches, medical and performance staff was essential
Arundale et al. (2015)[13]	USA	Clinical commentary	Text, tables and pictures	Football	Lower extremity	NR	The interval kicking programme increased kicking distance, volume, and intensity	Individualized, based on a player's response, soreness, feedback and injury	Coaches assisted the rehabilitation team in assessing passing, lofting, and shooting skills

Author (year)	Country	Sources of evidence	Description of OFR	Sport	Target condition	Sport level	Progression	Criteria for progression	Principles in the OFR and RTS process
Bien et al. (2015)[18]	USA	Clinical commentary	Text and pictures	NR	ACLR	NR	Targeted neuromuscular training that mimics sport-specific movements	Assessed movement quality (Functional Movement Screen and Y Balance Test) and psychological readiness	Used external cues with feedback to improve hip/knee flexion for better force absorption and hamstring activation
Bizzini et al. (2012)[14]	Switzerland	Clinical commentary	Text, tables, figures, pictures and videos	Football	ACLR	NR	4-stage rehabilitation; field drills were gradually intensified and progressed to team practice	No pain/swelling, symmetrical ROM, neuromuscular control, LSI strength >95% and hop >90%	Individual physiotherapy coaching and close team collaboration were vital for rehabilitation
Buckthorpe et al. (2019)[9]	Italy	Clinical commentary	Text, tables, figures, pictures and videos	Football	ACLR	NR	5-stage rehabilitation: (1) linear movement, (2) multi-directional movement, (3) soccer-specific technical skills, (4) soccer-specific movement, and (5) practice simulation	The criteria for starting OFR were: no instability, minimal pain, no effusion, full ROM, LSI strength <20%, be able to run at 8 km/ h for >10 min	The programme facilitated communication, planning, control, and safety in RTS; used GPS
Capin et al. (2017)[23]	USA	Clinical commentary	Text, tables and video	Ice hockey	ACLR	NR	A 4-phase, on-ice skating progression with objective criteria to initiate each phase then gradual return to play	RTS: ≥9 months after ACLR and physician clearance without increase in effusion or soreness; ≥90% quadriceps and hip strength; ≥90% LSI on hop tests; ≥90% PROMs	The coach could assist when puck handling progressed to passing and shooting
Della Villa et al. (2020)[25]	Italy	Book chapter	Text, tables, and pictures	Basketball	ACL/ACLR	NR	A 5-stage on-parquet programme; 4 pillars: movement quality, conditioning, skills, and load	The criteria for starting OFR were: good movement quality, no instability, minimal pain, no effusion, full ROM, strength <20% between limbs, be able	Personalized, progressive rehabilitation focusing on neuromuscular function under rehabilitation team supervision

Author (year)	Country	Sources of evidence	Description of OFR	Sport	Target condition	Sport level	Progression	Criteria for progression	Principles in the OFR and RTS process
								to run at 8 km/ h for >10 min	
D'Onofrio et al. (2023)[44]	Italy	Technical report	Text and tables	Football	ACL/ACLR	Elite	The interval 15-step kicking programme increases kicking distance, passing techniques, and ball speed	Progression individualized based on clinical status, symmetry, technical quality and multi-disciplinary decision	Optimal outcomes in the RTS process required interprofessional cooperation and communication
Forelli et al. (2023)[19]	Italy	Clinical commentary	Text, tables, figures and pictures	Football	ACL	NR	A velocity-based return to sprint programme	Gradual, tailored sprint return based on force–velocity and acceleration profiles	Clinicians tailor their interventions using GPS
Fort-Vanmeerhaeghe et al. (2021)[42]	Spain	Descriptive framework	Text, tables, figures and video	Team sports; basketball	ACLR	NR	A 2-stage framework: (1) general recovery; (2) RTC, which occurs on a continuum; tasks on 6 levels	Progressed in a personalized way when the athlete tolerates the loads	Interdisciplinary (technical and medical teams) collaboration was essential for successful RTC
Giannakis et al. (2018)[47]	Australia	Literature review	Text, tables and video	NR	ACLR	NR	At 16–22+ weeks post-ACLR to develop strength, explosive power, speed, COD, agility, muscular endurance	Individualized; RTS criteria: no pain or swelling, full ROM, ≥85% quadriceps and hamstring strength, ≥85% LSI on hop tests, tolerate sport-specific activities; RTS after >7 months	NR
Joreitz et al. (2017)[26]	USA	Book chapter	Text, tables and figures	NR	ACLR	NR	5 phases: running, agility drills, jumping, hopping/cutting/ pivoting, and return to participation	Based on strength, neuromuscular control, and mastery of tasks; be able to run 2 miles without pain, swelling, or deviations	Agility drills progressed under supervision of a physiotherapist or athletic trainer; tests with minimal equipment
Kyritsis et al. (2015)[27]	Qatar	Book chapter	Text, tables, figures and pictures	Football	ACLR	NR	The Aspetar approach in 3 phases: running, OFR functional	OFR training criteria: no pain or swelling, full ROM, ≥75% muscle strength, run for 45–60	The OFR described can be used by physiotherapists as a guideline

Author (year)	Country	Sources of evidence	Description of OFR	Sport	Target condition	Sport level	Progression	Criteria for progression	Principles in the OFR and RTS process
							training, and OFR sports-specific training before returning to team training	min with proper alignment, good knee control during balance and hops; psychological readiness	
Noyes et al. (2019)[28]	USA	Book chapter	Text, tables, figures and pictures	Football and basketball	Serious knee injuries, ACLR	NR	Sportsmetrics soccer and basketball training programmes, designed to restore strength, balance, proprioception, and neuromuscular control	Completed a running and agility programme and a basic plyometric training programme before starting with the Sportsmetrics programmes	Cost-effective field tests; expert verbal cues combined with video feedback to improve performance
Powell (2017)[29]	USA	Book chapter	Text, tables and pictures	Any sport	Lower extremity	NR	4 phases: progressively more difficult activities specific to the demands of the sport	Assessed stability, control, balance, symmetry, compensations, and confidence before progression with several specific functional performance tests	Physiotherapist or athletic trainer assessed the sprinting technique and the tests before progression
Sclafani and Davis (2016)[15]	USA	Clinical commentary	Text, tables and pictures	Rugby	Lower extremity	NR	Controlled movements, agility drills, diagonal and multi-planar movements; rehabilitation tailored to the position played, the level of the athlete, and the type of injury	OFR training criteria: no pain or swelling, full ROM, ≥85% extremity and core strength, run for >20 min, trunk and knee control, 90% single limb stability	Needed a multi-disciplinary team using clinician- or coach-led drills while monitoring symptoms, movement, performance, and psychology
Taberner et al. (2019)[48]	UK	Editorial	Text, tables, figures and video	Football	Lower extremity, ACL	Elite	Control–chaos continuum model	Hop and strength tests; emphasized quantitative load progression and qualitative movement in competition	Used GPS and force platform

Author (year)	Country	Sources of evidence	Description of OFR	Sport	Target condition	Sport level	Progression	Criteria for progression	Principles in the OFR and RTS process
Taberner et al. (2025)[20]	UK	Clinical commentary	Text and figures	Football	Lower extremity, ACL	Elite	Control–chaos continuum model	A ~70% success rate in drill-related outcomes; evaluated mental and physical demands	Integrating coach input into drills enhances collaboration and speeds RTS by creating realistic problem-solving scenarios
Taberner et al. (2025)[22]	UK	Clinical commentary	Text and figures	Football	Lower extremity, ACL	Elite	Control–chaos continuum model	Used running load and cardiovascular response data to guide training adjustments; no pain or swelling (rated <2/10)	Close collaboration with coaching staff was essential
Taberner et al. (2023)[21]	UK	Clinical commentary	Text, tables and figures	Basketball	Any	Elite	Control–chaos continuum model	Used different strength and hop tests	Important to integrate coaching staff
Verstegen et al. (2012)[16]	USA	Clinical commentary	Text, tables, figures and video	American football	ACLR	NR	3 phases: from simple to complex	No pain or swelling, full ROM, no instability, appropriate quadriceps strength, a Functional Movement Screen test score ≥14	Healthcare professionals working together must know sport-specific movements and demands
Waters, E. (2012)[17]	USA	Clinical commentary	Text, figures pictures and video	Basketball	ACLR	High school to elite level	3 phases: the final phase focuses on strength, power, endurance, and basketball-specific agility	Phase 3: full knee extension, ≥135° flexion, ≥90% strength, ≥ 8% cutting and landing skills, and muscular and cardiovascular endurance	Team staff offer psychological insight; match decisions should be collaborative

ACL, anterior cruciate ligament; ACLR, anterior cruciate ligament reconstruction; COD, change of direction; GPS, global positioning system; LSI, limb symmetry index; NR. not reported; OFR, on-field rehabilitation; PROM, patient-reported outcome measure; ROM, range of motion; RTC, return to competition; RTS, return to sport.

Table S2 Five case report studies (level of evidence IV–V) reporting on-field rehabilitation exercises applicable for athletes with ACL injury

Author (year)	Country	Participants	Description of OFR	Sport	Sport level	Progression	Criteria for progression	Principles in the OFR and RTS process
Bibby and Tripodi (2021)[30]	Australia	A 15-year-old female, ACL injury left knee, 26.5 weeks rehabilitation	Text, tables and pictures	Team sports (netball, basketball, and tennis)	NR	A comprehensive 4-stage rehabilitation management plan; phase 3: sport-specific skills (agility, change of direction, cutting and coordination); phase 4: RTS; progressing from restricted to unrestricted training and reintroducing all required skills	Each phase was inclusive of goals to achieve, rehabilitation exercises, functional testing, and criteria for the patient to pass before progressing with rehabilitation, e.g. achieving strength and power, LSI >90%, adequate patient-reported outcomes and psychological readiness score	Treatment (non-ACLR) was planned collaboratively by the sports physician, patient, parents, osteopath, and an external sports physiotherapist; the patient had regular osteopath check-ins for strength testing, exercise programming and biomechanical analysis (cutting and change of direction); patient used a training diary
Cross (2022)[31]	Australia	A 22-year-old male, ACLR left knee, QC graft + LET, 364 days rehabilitation	Text, figures, tables, pictures and videos	Australian football	Elite	5 key phases: acute recovery, muscular control and coordination, proprioception and agility, sport-specific skills and RTS; control–chaos continuum model	Side difference less than; QC circumference ≤1 cm, isokinetic strength LSI <10% in QC and HS and 3 RM leg press <10%; psychological readiness	Key criteria for progression were developed by the rehabilitation team in conjunction with the strength and condition team; used GPS
Lehane (2020)[32]	Australia	A 21-year-old male, ACLR left knee, HS graft, 345 days rehabilitation	Text, figures, tables, pictures and videos	Rugby	Elite	Control–chaos continuum model	Criteria based (not specified) and not time dependent	Technical coaches, medical, and athletic performance staff guide basic skill components and contact training progression; used GPS
Mitchell et al. (2023)[33]	UK	A 26-year-old male, ACLR right knee, four-strand autologous HS (semitendinosus and gracilis) graft from the contralateral limb, medial meniscus suture/ectomy 8 months after ACLR, 10.5 months rehabilitation	Text and figures	Football	Elite	9 phases: return to performance pathway with the last 4 phases on grass; the on-field sport-specific criteria utilizing the control–chaos continuum model	Structured phases with exit criteria; progression based on objective performance measures rather than time; readiness assessed via strength, running, hop tests and self-rated psychological readiness	The RTS had a clear plan and a progressive pathway for the player, members of the multi-disciplinary team and coaches to follow; used GPS
Taberner et al. (2020)[34]	UK	A 24-year-old female, ACLR left knee, four-strand autologous HS	Text, figures, tables,	Football	Elite	Control–chaos continuum model with return to performance	Objective measures included hop tests, isometric hip strength, isokinetic strength	Successful RTS is supported by objective preinjury data, clinical reasoning, and shared decision-

Author (year)	Country	Participants	Description of OFR	Sport	Sport level	Progression	Criteria for progression	Principles in the OFR and RTS process
		(semitendinosus and gracilis) graft, 13–14+ months rehabilitation	pictures and videos			pathway, which aids a progressive return to team training and competition	tests, daily assessment of pain (numerical rating scale) and degree of joint effusion	making; rehabilitation coach coordinated with coaching staff during the modified training reintegration; used GPS

ACL, anterior cruciate ligament; ACLR, anterior cruciate ligament reconstruction; GPS, global positioning system; HS, hamstring; LET, lateral extra-articular tenodesis; LSI, limb symmetry index; NR, not reported; OFR, on-field rehabilitation; QC, quadriceps; RM, repetition maximum; RTS, return to sport.

Table S3 Nine studies with a prospective cohort, RCT or case series design reporting on-field rehabilitation exercises applicable for athletes with ACL injury

**Author (year) country**	**Sources of evidence/level of evidence (I–V)**	**Participants**	**Description of OFR**	**Intervention/aim**	**Results/conclusion**	**Principles in the OFR and RTS process**
Arundale et al. (2018)[45] USA	Single-blinded randomized controlled trial/II	79 (49% women) athletes, 3–9 months after ACLR participating in cutting, pivoting, and jumping sports before ACLR and not returned yet, median age, 18.7 years (range, 13–54 years)	Text, tables and figures	Examine the effects of knee function and PROMs using a secondary ACL prevention programme that included 10 (5 weeks) sessions of progressive strengthening, agility training, plyometrics (SAP), and other components of current primary prevention protocols, with (1) perturbation training (SAP + PERT group) and (2) without PERT (SAP group)	Improvements in hop test limb symmetry and PROMs among predominantly level I athletes, regardless of groups; men improved in QC limb symmetry, whereas women did not; the 10-session programme is potentially beneficial for RTS after ACLR; women may need additional QC strengthening	The programme was supervised by a physiotherapist; emphasis on landing techniques; inclusion criteria: ≥80% QC symmetry, minimal effusion, no pain, full ROM, successful completion of a running progression programme
Della villa et al. (2012)[36] Italy	Case series/IV	50 (12% women) competitive football players with ACLR; age 23 ± 6 years (range, 16–37 years); the mean start of OFR was 90 ± 26 days after ACLR	Text and tables	To describe the recovery process and the times for reaching clinical and functional goals by following a specific rehabilitation protocol focused on the final phase of functional recovery	Improvement in PROMs, QC and HS muscle strength and aerobic threshold; adding OFR to the traditional protocols after ACLR may safely lead to complete functional recovery in football players	Criteria for starting OFR were: no instability, minimal pain, no effusion, full ROM, strength <20% between limbs, and ability to run at 8 km/h for >10 min; OFR comprised 5 progressive phases; advancement required symptom-free, coordinated exercise (based on function, not time); an athletic trainer supervised the programme
Joreitz et al. (2020)[38] USA	Case series/IV	43 (44% women) participants with ACLR, age 27.2 ± 8.5 years (range, 16–50 years) with the goal to RTS	Text and tables	To evaluate RTS and reinjury rates following the use of a criterion-based rehabilitation protocol with a final RTS test	21 participants (49%) completed the rehabilitation, including passing RTS tests and 19 participants (44%) were available for the 2-year follow-up; at the 2-year follow-up, 16 of the 19 (84%) returned to their preinjury level of sports; the mean time from ACLR to RTS clearance was 10.6 ± 4.4 months; only one reinjury at the 2-year follow-up	Criterion-based progression through 5 phases (ADLs, running, agility, jumping, and cutting); clearance: strength LSI ≥90%, hop/balance tests, task mastery, psychological readiness; the agility progression was initially supervised by a physiotherapist or athletic trainer to monitor quality impairments; minimal equipment required
Moksnes et al.	Prospective cohort/II	43 (67% women) with ACLR 6 months earlier,	Text, tables and videos	To assess the implementation, limited efficacy, and acceptability	Hop and QC power performance improved significantly; athletes	The BEAST tool was designed in collaboration with athletes,

Author (year) country	Sources of evidence/level of evidence (I–V)	Participants	Description of OFR	Intervention/aim	Results/conclusion	Principles in the OFR and RTS process
(2021)[39] Norway		nonprofessionals participating in pivoting sports (football, handball, basket and floorball), median age, 19 years (range, 15–31 years)		of the BEAST tool, a rehabilitation and RTS decision tool after ACLR	believed the BEAST rehabilitation would facilitate RTS and reduce injury risk	primary care and sports physiotherapists, and coaches; criteria to progress: no effusion or pain, sport-specific training status, hop and QC test results, and biological healing of the graft (minimum 10 months after ACLR to play matches)
Nyland et al. (2020)[41] USA	Prospective cohort/II	150 (45% women) athletically active amateur teenagers or adults (mostly football, basketball and American football players) with ACLR a mean of 7 months earlier, age 20.3 ± 7.2 years	Text and tables	To determine the efficacy of an 8-week, twice-weekly supplemental RTS bridge training programme before unrestricted sports participation for improving PROMs and the reinjury rate; the programme focused on whole-body neuromuscular control through strength/power, sprint and agility training with sport-specific task performance	The RTS bridge programme improved PROMs, and 1.3% non-contact ipsilateral and 2.7% non-contact contralateral knee injury rates were low; 100% RTS by 9.1 ± 2.5 months after ACLR, 84% to the same or better performance level	All participants had to meet test criteria before unrestricted sports: ≥90% LSI on hop tests and strength, PROMs ≥85%, no limb favouring or effusion; results shared with surgeon and trainer; after 2 weeks of supervised full practice without issues, the athlete was cleared for unrestricted play
Picinini et al. (2025)[35] Italy	Case series/IV	100 amateur and elite male football players with ACLR cleared from the surgeon and sports medicine physician to RTC, age 23.2 ± 5.8 years	Text and tables	To document the activity and workloads completed by football players during OFR after ACLR and completing their indoor rehabilitation and to establish their RTC outcomes	Follow-up: 97 players (median 2.3 years post-ACLR); 84% RTC (elite 100%, amateurs 80%); ACL reinjury: 10%; OFR improved readiness, but workloads remained below match demands	Progressed through 5-phase OFR and the control–chaos continuum model focusing on movement quality, conditioning, football skills, and load management with a criteria-based approach; rehabilitation coaches supervised the OFR; used GPS
Stathas et al. (2024)[46] Greece	A prospective block-randomized controlled-parallel trial/Ib	76 male elite football players with a lower extremity injury. e.g. ACL, age 26.3±3.6 years (range, 17–40 years)	Text, tables, figures and video	To evaluate the effectiveness (time needed to return to team training participation and subsequent injuries) of an accelerated (early initiation) OFR framework for RTS compared with a traditional OFR framework for a late injury recovery phase	The evaluated accelerated OFR framework reduced the time a player needed to RTS compared with a traditional OFR framework with a very low risk of reinjury	Progressed through 5-phase OFR; all participants completed all stages of rehabilitation under the guidance of the author's team who were responsible for monitoring criteria (pain, effusion and ROM) to progress; communication among the sports physician, physiotherapists, and participants was continuous; used GPS

Author (year) country	Sources of evidence/level of evidence (I–V)	Participants	Description of OFR	Intervention/aim	Results/conclusion	Principles in the OFR and RTS process
Walker et al. (2024)[37] Australia	Case series/IV	60 participants (30% women) with ACLR median of 7.5 months earlier, participating in rugby, basketball, football, American football, netball, etc.; median age, 20 years (range, 13–36 years)	Text, figure and tables	To investigate the effectiveness (strength, hop tests, running T test, PROMs) of a physiotherapist-led group 8-week rehabilitation programme on functional outcomes and PROMs after ACLR	There were improvements in all RTS criteria tests (>90% LSI in isometric strength and hop tests, running T test <12 s, IKDC >90% and ACL-RSI >80%) after completing the rehabilitation programme, but few participants (8%) passed all RTS criteria; 85% returned to their previous sport level	Criteria to be included: minimal pain (<3/10), no swelling, symptom-free straight-line running (>70% effort), and tolerate strength and jump training without increased symptoms; progression guided by performance and tolerance
Zhang et al. (2024)[40] China	Prospective cohort/II	50 high school male football players with various injuries, age 16.4 ± 1.3 years (range, 15.5–17.7 years)	Text and tables	To determine whether short-term HIIT and MICT improve VO_2 peak, exercise duration, AT, anaerobic power, fatigue index, and lower limb strength in athletes who had almost completed their rehabilitation and were about to RTS; the players underwent a 12-session intervention training programme that lasted 4 weeks	Short-term HIIT and MICT were effective methods for improving fitness in young male football players preparing to RTS after injury; HIIT was slightly more effective than MICT in enhancing aerobic and anaerobic fitness as well as strength	HIIT: 3 weekly sessions for 4 weeks; 30 s sprint/30 s jog, 5 reps per set, 4 sets (~1 h). HR >80% work, >50%–65% rest, monitored electronically; MICT: 12 sessions over 4 weeks; continuous trekking at 65%–75% HRmax, two 20-min sets with 10-min rest, HR monitored

ACL, anterior cruciate ligament; ACLR, anterior cruciate ligament reconstruction; ACL-RSI, Anterior Cruciate Ligament Return to Sport after Injury; ADL, activity in daily living; BEAST, better and safer return to sport; GPS, global positioning system; HS, hamstring; PERT, perturbation training; QC, quadriceps; HIIT, high-intensity interval training; HR, heart rate; MICT, moderate-intensity continuous training; LSI, limb symmetry index; IKDC, International Knee Documentation Committee 2000 subjective knee evaluation questionnaire; OFR, on-field rehabilitation; PROM, patient-reported outcome; RTC, return to competition; RTS, return to sport; ROM, range of motion; VO2, volume of oxygen uptake
